# Characterization of a *Salmonella abortus equi* phage 4FS1 and its depolymerase

**DOI:** 10.3389/fvets.2024.1496684

**Published:** 2024-11-25

**Authors:** Jianchao Zhao, Jiayu Wang, Can Zhang, Shouzhen Xu, Huiying Ren, Ling Zou, Jing Ma, Wenhua Liu

**Affiliations:** College of Veterinary Medicine, Qingdao Agricultural University, Qingdao, China

**Keywords:** *S. abortus* equi, phage 4FS1, depolymerase, biofilm, polysaccharides

## Abstract

The significant economic losses caused by *S. abortus equi* in donkey husbandry have increased interest in exploring the potential of phages and their enzymes as control strategies. In this study, a *S. abortus equi* phage, designated 4FS1, was isolated from sewage at a donkey farm. Transmission electron microscopy (TEM) revealed a typical icosahedral head and a long, non-contractile tail. It exhibited a short latent period of 20 min and a burst size of 160 plaque-forming units (PFU) per cell. It demonstrated a broad host range, infecting 36 out of 60 *salmonella* strains, with an optimal multiplicity of infection (MOI) of 0.01 for *S. abortus equi* S1. The phage titer remained stable at 10^9^ PFU/mL between 37°C and 50°C and exceeded 10^8^ PFU/mL at pH from 5.0 to 10.0. After 1 h of UV exposure, the titer remained at 10^7^ PFU/mL and showed no significant variation across NaCl concentrations from 2.5 to 15%. The genome of phage 4FS1 consists of a 42,485 bp linear double-stranded DNA molecule with a G + C content of 49.07%. Of the 56 predicted open reading frames (ORFs), 32 were functional annotated, with no virulence or drug resistance genes identified. ORF36 was predicted to encode a depolymerase responsible for endorhamnosidase activity. Recombinant expression of the Dpo36 protein in prokaryotes significantly reduced biofilm formation and removal. Combined with healthy donkey serum, Dpo36 inhibited bacterial growth *in vitro* and enhanced the survival rates of mice infected with *S. abortus equi.* These findings highlight the promising potential of phages and their depolymerases as novel therapeutic agents against *S. abortus equi*.

## 1 Introduction

*Salmonella abortus equi* is a prominent pathogen known to cause abortion in equidae (horses, donkeys, and mules) (1). The infection is widespread and well-established within equine populations (2). Donkeys of all ages are susceptible to *S. abortus equi*, with primiparous dams and foals being at the highest risk (3). Many infections are asymptomatic, but abortions can occur at any gestational age, particularly during the late stages of pregnancy. Secondary infections can lead to serious systemic manifestations, such as fever, purulent, and bloody vaginal discharge. Uterine infection can persist for extended periods, ultimately causing infertility or recurrent abortions in mares over several years (4–6).

Bacterial polysaccharides play a crucial role in biofilm formation, serving as a protective barrier against phage infection, enhancing antibiotic resistance, and aiding bacteria evasion of the immune system (7, 8). These polysaccharides enable bacteria to readily attach to and persist on sinks and floors, rendering them resistant to conventional cleaning methods and promoting bacterial growth (9, 10).

The ability of phages to multiply within bacteria and induce lysis has emerged as a promising biocontrol strategy for managing bacterial infections (5). The use of lytic phages as a therapeutic alternative to antibiotics has gained significant interest, especially with the rising prevalence of antibiotic-resistant bacteria in the livestock industry (11). While phages have the potential to eradicate drug-resistant *Salmonella*, the presence of extracellular polysaccharides can hinder phage attachment to the host receptor (12). Phages overcome this barrier by producing depolymerases that target glycosidic linkages within polysaccharides. Depolymerases have different types, including endorhamnosidase, sialidases, levanases, xylosidases, dextranases, hyaluronidases, peptidases, and others (13). This enzymatic action breaks the polysaccharide structure, releasing repeating polymer units and facilitating phage infection of bacterial cells (14, 15). Researches have demonstrated the efficacy of phage depolymerases in disrupting and inhibiting biofilm formation (16, 17). When combined with serum, phage depolymerase exhibits potent bacteriostatic and bactericidal activities both *in vitro* and *in vivo* settings, suggesting their potential for controlling pathogenic bacteria (18, 19).

This study focuses on characterizing a *S. abortus equi* lytic phage 4FS1 and its depolymerase. We investigated the biological characteristics of 4FS1 and its depolymerase to better understand the potential applications of phages and depolymerases for combating *S. abortus equi* infection.

## 2 Materials and methods

### 2.1 Samples and bacteria strains

Donkey sera utilized in this study were obtained from farms in Shandong, China. Sixty *Salmonella* strains, including *S. abortus equi* S1 identified through sequencing and exhibited multidrug resistance were provided by the Veterinary Microbiology Laboratory at Qingdao Agricultural University. All strains were cultured in Luria-Bertani (LB) medium at 37°C.

### 2.2 Isolation and purification of phages

Sewage samples were collected from a donkey farm located in Shandong, China to isolate phages using the double-layer agar method with *S. abortus equi* S1 as the host strain (20). The samples were centrifuged at 12,000 g for 10 min, followed by filtration through a 0.45 μm filter. The resultant supernatant was subsequently mixed with an equal volume of *S. abortus equi* S1 bacterial suspension (10^9^ CFU/mL) in fresh LB broth and incubated at 37°C overnight to enrich phages. Centrifugation and filtration were performed again, then the filtrate was mixed with an equal volume of *S. abortus equi* S1 bacterial suspension (10^9^ CFU/mL, 100 μL) and incubated at 37°C for 5 min. Subsequently, 5 mL of 0.9% molten agar was poured onto a Nutrient Agar (NA) plate and incubated at 37°C overnight. A single plaque was selected and purified by the double-layer agar method until the shape and size were consistent.

### 2.3 Microscopy observation

Morphology of the isolated phage was observed as a previously established method with slight modification (21). Briefly, the phage suspensions (≥10^9^ PFU/mL) of 20 μL were incubated on the carbon-coated grid for 15 min, and then stained with 1% phosphotungstic acid for 5 min. Finally, the grid was air-dried at 70°C in the dark, and phages were observed under an 80 kV accelerated voltage TEM (Hitachi, Japan).

### 2.4 Multiplicity of infection (MOI) and one-step growth curve

The MOI and one-step growth curve of the phage were established as previously described with minor modifications (22). Specifically, Phage and host strain of *S. abortus equi* S1 (10^6^ CFU/mL) were co-cultured at 37°C for 4 h based on various MOIs (0.001, 0.01, 0.1, 1, and 10). The phage titer was assessed using the double-layer agar method to determine the optimal MOI. For the one-step growth curve, *S. abortus equi* S1 (10^7^ CFU/mL) was mixed with the phage proliferation solution at an MOI of 10 and incubated for 5 min at 37°C. Following centrifugation at 12,000 g for 5 min, the supernatant was discarded, and the precipitate was washed twice with LB broth, then resuspended in LB broth and incubated at 37°C with agitation. Samples of 20 μL were collected at 5-min intervals during the first hour, 20-min intervals during the second hour, and 30-min intervals during the third hour. These samples were then centrifuged at 12,000 g for 1 min, and the supernatants were used for phage titer determination via the small drop plaque assay method (23). The burst size was determined by calculating the ratio of the final number of released phage particles to the initial number. The experiments were conducted in triplicate.

### 2.5 Environmental stability of the phage

Thermal stability, pH sensitivity, and UV sensitivity of the phage were evaluated following previously established protocols, with minor modifications (24). Specifically, for thermal stability, the phage suspension was incubated at temperatures from 37°C to 80°C, with samples taken at 20, 40, and 60 min for titer analysis. For pH stability, the phage suspension was incubated across pH levels 2–13 at 37°C for 1, 2, and 3 h. Phage titers were determined using the double-layer agar method. For UV sensitivity, the phage suspension was exposed to UV (30 W, 26.23 μw/cm^2^) at 40 cm for 120 min, with titers measured every 10 min using the double-layer plate method. The stability of phage 4FS1 with NaCl was assessed using a modified method (25). Briefly, a 5 mL phage suspension (10^9^ PFU/mL) was mixed with equal volumes of NaCl solutions at concentrations from 2.5 to 15%. After 4 h at 37°C, the phage titer was quantified using the small drop method. Experiments were conducted in triplicate.

### 2.6 Host range and efficiency of plating (EOP) measurement

The host range of phage was assessed using plaque assays conducted on 60 Salmonella strains. In brief, each strain was incubated overnight at 37°C, and then 100 μL of each bacterial culture was mixed with 3 mL of soft LB agar (0.7%, w/v) and poured onto NA agar plates (1.5%, w/v). After solidification, 10 μL of phage filtrate (~10^9^ PFU/mL) was added, and the plates were incubated overnight at 37°C. Phage infectivity was assessed by observing plaque morphology, turbidity, and relative efficiency of plating (EOP) using the small drop plaque assay. Specifically, plates with various strains were incubated with 20 μL of phage at various dilutions (10^1^ ~ 10^7^) and then incubated at 37°C for 4 h. EOP values were calculated by comparing the phage titer of each tested Salmonella strain to the reference strain S. abortus equi S1, whose EOP value was considered as 1 (26). EOP values were categorized as high (EOP ≥ 0.5), medium (0.1 ≤ EOP < 0.5), low (0.001 < EOP < 0.1), and none (EOP ≤ 0.001) based on lytic capacity (27).

### 2.7 Genomic DNA extraction, sequencing, and bioinformatic analysis

Genomic DNA extraction, sequencing, and bioinformatics analysis of the phage were conducted following established protocols (28). The genomic DNA of the phage was extracted utilizing a viral genomic DNA/RNA extraction kit from Tiangen Biochemical Technology Co., LTD. in Beijing, China. Sequencing services were provided by BIOZERON Biotechnology Co., LTD. in Shanghai, China. Raw sequence reads were processed using the NGS QC Tool kit (29). The complete sequence of the phage was annotated using RAST^1^ and GeneMark^2^ (30, 31). Predicted ORFs were validated through the utilization of the online BLASTP tool available at http://www.ncbi.nlm.nih.gov/BLAST. Putative transfer RNA (tRNA)-encoding genes were identified using tRNAscan-SE^3^ (32). The online tool PHASTER^4^ was employed for the examination of lysogenic genes within the genome, while the CGE server^5^ was utilized for the analysis of phage resistance genes and virulence genes. Phylogenetic trees based on the entire genome and terminase large subunit were generated using the Neighbor-joining method with default parameters in MEGA 5.0 (33).

### 2.8 Homology modeling and active site analysis of the putative depolymerase

A phage-related depolymerase gene, ORF36, was identified through sequence analysis. Using the Swiss Model platform,^6^ its amino acid sequence homology was compared. Subsequently, the structural characteristics of the ORF36 protein were visualized with PyMOL software, and its spatial arrangement was further analyzed via CD-search.^7^

### 2.9 Cloning, expression, and purification of the putative depolymerase

Following standard protocols, the putative depolymerase of Dpo36 gene (ORF36) was cloned and expressed (34). Briefly, the target gene was amplified by PCR using a specific forward primer (5′-ctcggtaccctcgagggatccATGTCACTTAATGATTTACAGATAGCT-3′) and a reverse primer (5′-agcagagattacctatctagaTTATGCCAAAGTTAATCTTGTATAGCTT-3′) designed with CE Design software. The recombinant plasmid pColdTF-Dpo36 was then generated through homologous recombination of the PCR product with the pColdTF vector. Then, the recombinant plasmid was transformed into *E. coli* BL21 (DE3) and expressed with 0.1 mM IPTG at 16°C for 16 h. After washing twice with sterile PBS, the bacterial suspension was ultrasonically disrupted, centrifuged, and the supernatant was collected for purification. The recombinant protein was purified using a Ni-NTA gravity column and eluted with imidazole concentrations of 50 mM, 100 mM, 200 mM, and 500 mM. Finally, the eluted protein underwent dialysis in PBS at 4°C for 24 h, with PBS changes every 6 h. To determine whether the pColdTF vector affected the protein activity, the recombinant protein was digested with HRV 3C protease at 4°C for 16 h according to the instructions, and protein presence was confirmed through SDS-PAGE analysis.

### 2.10 Thermal and pH stability of Dpo36

The thermal and pH tolerance of Dpo36 were determined as previously described (35). For thermal tolerance, Dpo36 (100 μg/mL) was incubated for 60 min at temperatures ranging from 20°C to 70°C, then cooled on ice for 2 min. Activity was measured by comparing the lowest active dilution of heat-treated and untreated samples. For pH tolerance, Dpo36 (100 μg/mL) was incubated for 60 min across a pH range of 3 to 12, and activity was similarly assessed by comparing treated and untreated samples.

### 2.11 Degradation of polysaccharides by Dpo36

The polysaccharide degradation activity of Dpo36 was determined using the dot plate method (36). Briefly, 200 μL of *S. abortus equi* S1 was mixed with soft LB agar (0.7%, w/v) and then spread on a NA agar (1.5%, w/v) plate. Purified Dpo36 was applied in 2 μL spots at concentrations of 1,000, 100, 10, 1 and 0.1 μg/mL with each sample tested in triplicate.

To evaluate the effect of Dpo36 degradation on bacterial surface polysaccharides, polysaccharides were extracted from *S. abortus equi* S1 using the hot water-phenol method (37). As shown in Table 1, the experimental groups were subjected to SDS-PAGE electrophoresis to assess the impact of Dpo36 on surface polysaccharides, followed by silver staining after 1-h incubation at 37°C.

**Table 1:** Degradation scheme of Dpo36 on the polysaccharides extracts.

Groups	Polysaccharides (μL)	Dpo36 (100 μg/mL, μL)	PBS (μL)	Total volume (μL)
1	200	0	200	400
2	200	200	0	400
3	200	200 (inactivated)	0	400
4	0	200	200	400

To further evaluate the effect of depolymerase on the *S. abortus equi* S1, the experiment was conducted using slightly modified established protocols (35). *S. abortus equi* S1 at 10^9^ CFU/mL was suspended in PBS, treated with 100 μg/mL Dpo36, and incubated at 37°C for 4 h, with a PBS-treated negative control. A copper mesh was used to prepare samples of bacteria, and their morphology was examined under TEM (Hitachi, Japan) after they were washed twice with PBS and fixed overnight with 2.5% glutaraldehyde.

### 2.12 Biofilm degradation activity of Dpo36

To assess the impact of Dpo36 on biofilm formation, 100 μL of *S. abortus equi* S1 (10^6^ CFU/mL) and various Dpo36 concentrations (100, 10, 1, 0.1, 0.01 μg/mL) were added to a 24-well plate, with PBS as a control. After a 24-h incubation at 37°C, wells were rinsed with PBS, and biofilm content was quantified using crystal violet staining (38).

To determine the effect of Dpo36 on established biofilms, *S. abortus equi* S1 (10^6^ CFU/mL) was cultured for 24 h in a 24-well plate to form biofilms, then washed with PBS. Dpo36 was added at various concentrations (100, 10, 1, 0.1, 0.01 μg/mL) with PBS as a control. After incubation at 37°C for 2 h, the liquid was removed, wells were rinsed with PBS, and biofilm content was measured by crystal violet staining intensity.

### 2.13 Bacterial inhibition by Dpo36 combined with serum *in vitro*

The Bacterial inhibition by Dpo36 combined with serum was assessed following previously established protocols (39). We evaluated the effects of Dpo36 and serum from healthy donkeys on bacterial activity. The inhibitory activity was measured by mixing Dpo36 with serum using the dot plate method, where a mixture of 200 μL of *S. abortus equi* S1 and soft LB agar (0.7%, w/v) was applied to a NA agar plate (1.5%, w/v). Purified Dpo36 at concentrations of 1,000, 100, 10 and 1 μg/mL, along with the ratio of donkey serum at 100, 50, 25, 12.5 and 6.25%, were added in 3 μL increments to a plate with inactivated serum (heated at 55°C for 30 min) as a negative control. The plate was then incubated at 37°C for 8 h.

To evaluate the impact on bacterial growth, a 96-well plate containing Dpo36 at 100, 10, and 1 μg/mL and donkey serum at 25 and 12.5% was used with *S. abortus equi* S1 (10^6^ CFU/mL). The plate was incubated at 37°C, and OD_600_ was measured over time to monitor bacterial growth.

### 2.14 Antimicrobial activity of Dpo36 *in vivo*

To evaluate the efficacy of Dpo36 against *S. abortus equi* S1 in mice, female KM mice were allocated into four groups of ten. The pre-treated group received a 200 μL intraperitoneal injection of Dpo36 (500 μg/mL) 1 h before being inoculated with 10^7^ CFU of *S. abortus equi* S1. The treated group was first inoculated with 10^7^ CFU of *S. abortus equi* S1, followed by an intraperitoneal injection of 200 μL Dpo36 (500 μg/mL) 1 h later. The challenged control group was administered a 200 μL intraperitoneal injection of *S. abortus equi* S1 at 10^7^ CFU for 1 h, followed by 200 μL of PBS. The control group was injected with 200 μL of PBS only. After injection, mice were evaluated using a modified 0–5 scale: 0 for death, 1 for prostration, 2 for closed eyes and eye secretion, 3 for lethargy and arched back, 4 for reduced movement and rough coat, and 5 for health (40). Survival rates were tracked for 7 days, after which mice were euthanized using CO_2_ and cervical dislocation. Three mice per group were selected for bacterial load assessment in the liver, spleen, and kidneys. Organs were collected, homogenized in 0.9% NaCl, plated on NA agar, and incubated at 37°C for 12 h. *S. abortus equi* colonies were then counted and reported as CFU/g. All groups had unrestricted food and water access and were kept in sanitary conditions throughout the 7-day test.

### 2.15 Statistical analysis

Experiments were performed at least three times, each in triplicate. Data are presented as means ± SD and analyzed with GraphPad Prism 6.02 (GraphPad Software, Boston, MA, USA). Group differences were assessed using one-way ANOVA with Tukey’s *post hoc* test, with significance set at *p* < 0.05.

## 3 Results

### 3.1 Morphology of phage

**Figure 1:**
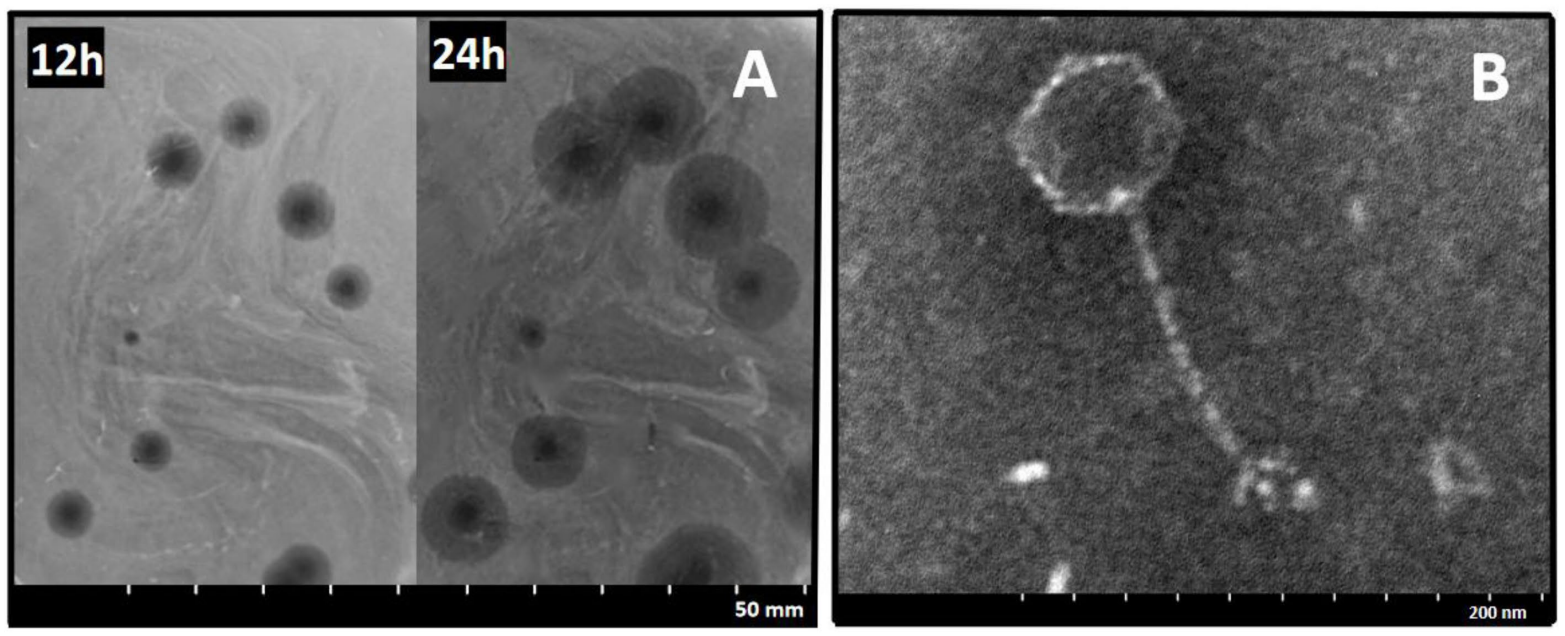
Morphology of phage 4FS1. **(A)** Plaques produced by phage 4FS1 on the lawn of *S. abortus equi* S1. **(B)** TEM picture of phage 4FS1. TEM image of phage 4FS1 with a regular icosahedral head (about 60 nm in diameter) and a long unretractable tail (about 120 nm in length).

A *Salmonella*-specific phage, 4FS1, was obtained from sewage and formed clear plaques with translucent halos on double-layer agar plates when infecting *S. abortus equi* S1 (Figure 1A). TEM images revealed its icosahedral head and long tail (Figure 1B).

### 3.2 MOI and one-step growth curve of phage 4FS1

**Figure 2:**
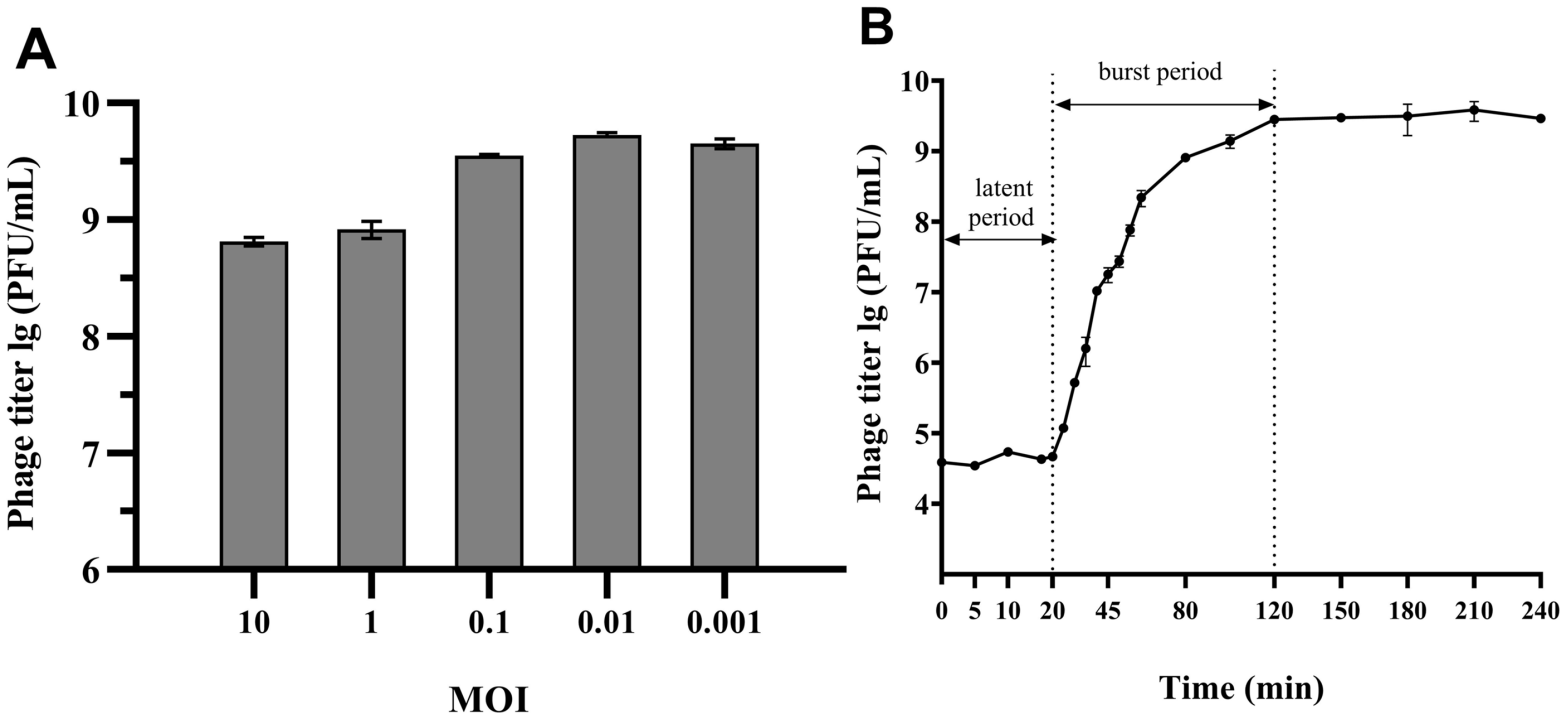
MOI and One-step growth curve of phage 4FS1. **(A)** The titers of phage 4FS1 were examined at various MOIs (0.001, 0.01, 0.1, 1, and 10). **(B)** The one-step growth curve of phage 4FS1. Phage 4FS1 was combined with *S*. *abortus equi* S1 at an MOI of 10. Burst size was calculated as the phage titer at the end of infection divided by the host bacteria titer at the beginning of infection. Data are presented as mean ± SD (*n *= 3).

Figures 2A,B demonstrated that MOI between 0.1 and 0.001 enabled phage 4FS1 to reach a peak titer of approximately 10^9^ PFU/mL. A one-step growth curve assay at an MOI of 10 revealed a 20-min incubation, a 100-min burst period, and a burst size of 160 PFU per cell for phage 4FS1.

### 3.3 Environmental stability of phage 4FS1

Supplementary Figure S1A showed that phage 4FS1 was stable below 50°C, with no significant titer change between 37°C and 50°C. The titer dropped from 10^9^ PFU/mL to 10^5^ PFU/mL after 20 min at 70°C and was inactivated at 80°C after 20 min. It maintained high activity (10^8^ PFU/mL) at pH 5–10 for at least 3 h, suggesting stability in this pH range (Supplementary Figure S1B). The titer remained at 10^7^ PFU/mL after 1 h of UV exposure (Supplementary Figure S1C). Supplementary Figure S1D illustrated that phage 4FS1 tolerated salt concentrations from 2.5 to 15.0% with no significant titer reduction after 4 h.

### 3.4 Host range and EOP

As shown in Table 2, phage 4FS1 exhibited lytic activity against 60% (36/60) of tested *Salmonella* strains, with varying EOP indicating differences in susceptibility.

**Table 2:** Lytic activity of phage 4FS1 against the tested *Salmonella* strains.

No.	Strain	EOP		Source	ST
1	*S. typhimurium* ATCC14028	0.100	Medium	Standard strain, Weifang, China (2021)	1
2	*S. enteritidis* 270	1.000	High	Duck feces, Weifang, China (2021)	2
3	*S. typhimurium* 297	–	–	Duck feces, Qingdao, China (2022)	3
4	*S. enteritidis* HIJ062	0.200	Medium	Duck feces, Qingdao, China (2021)	4
5	*S. enteritidis* JS074	0.040	Low	Duck liver, Yantai, China (2023)	5
6	*S. enteritidis*sd150	–	–	Dead chicken embryo, Qingdao, China (2023)	6
7	*S. enteritidis* HB167	0.700	High	Dead chicken embryo, Qingdao, China (2022)	7
8	*S. enteritidis* JS228	–	–	Chicken liver, Yantai, China (2021)	8
9	*S. enteritidis* S14-64	1.000	High	Chicken liver, Yantai, China (2020)	9
10	*S. enteritidis* S14-419	–	–	Chicken liver, Weifang, China (2022)	10
11	*S. enteritidis* S14-411	–	-	Chicken liver, Weifang, China (2024)	11
12	*S. enteritidis* S24	–	–	Chicken liver, Yantai, China (2021)	12
13	*S. typhimurium* CVCC542	0.016	Low	Standard strain, Qingdao, China (2023)	13
14	*S. typhimurium* CVCC533	0.048	Low	Standard strain, Yantai, China (2020)	14
15	*S. enteritidis* JC927	1.000	High	Chicken liver, Yantai, China (2018)	15
16	*S. enteritidis S*F1	–	–	Donkey feces, Weifang, China (2018)	16
17	*S. enteritidis S*F2	–	–	Donkey feces, Qingdao, China (2018)	17
18	*S. enteritidis S*F3	–	–	Donkey fecesDonkey feces, Jinan, China (2020)	18
19	*S. enteritidis S*F4	–	–	Donkey feces, Yantai, China (2021)	19
20	*S. enteritidis S*F5	–	–	Donkey feces, Qingdao, China (2021)	20
21	*S. enteritidis S*F6	–	–	Donkey fecesDonkey feces, Jinan, China (2021)	21
22	*S. enteritidis S*F7	–	–	Donkey feces, Weifang, China (2022)	22
23	*S. enteritidis S*F14	0.600	High	Donkey fecesDonkey feces, Jinan, China (2023)	23
24	*S. enteritidis S*248	0.150	Medium	Donkey feces, Qingdao, China (2019)	24
25	*S*. *abortus equi* S1	1.000	High	Donkey feces, Yantai, China (2020)	25
26	*S. typhimurium S*414	–	–	Chicken embryo, Qingdao, China (2017)	26
27	*S. typhimurium S*415	–	–	Pig fecesDonkey feces, Jinan, China (2017)	27
28	*S. typhimurium S*417	0.750	High	Pig feces, Weifang, China (2018)	28
29	*S. typhimurium S*419	–	–	Pig fecesDonkey feces, Jinan, China (2018)	29
30	*S. typhimurium S*430	0.020	Low	Pig fecesDonkey feces, Jinan, China (2018)	30
31	*S. typhimurium S*433	–	–	Pig feces, Qingdao, China (2019)	31
32	*S. typhimurium S*437	–	–	Pig fecesDonkey feces, Jinan, China (2020)	32
33	*S. enteritidis S*439	–	–	Pig feces, Yantai, China (2021)	33
34	*S. enteritidis S*443	0.020	Low	Pig fecesDonkey feces, Jinan, China (2021)	34
35	*S. enteritidis S*447	–	–	Pig feces, Yantai, China (2021)	35
36	*S. enteritidis S*449	0.017	Low	Pig feces, Qingdao, China (2021)	36
37	*S. enteritidis S*453	0.015	Low	Rabbit liver, Qingdao, China (2021)	37
38	*S. typhimurium S*423	0.150	Medium	Mouse uterusDonkey feces, Jinan, China (2022)	38
39	*S. typhimurium S*425	0.100	Medium	Mouse uterus, Qingdao, China (2023)	39
40	*S. typhimurium S*427	1.000	High	Mouse uterusDonkey feces, Jinan, China (2023)	40
41	*S. typhimurium* JH028	–	–	Donkey feces, Qingdao, China (2017)	41
42	*S. enteritidis S*3	–	–	Donkey feces, Qingdao, China (2023)	42
43	*S*. *abortus equi* A1	0.100	Medium	Donkey feces, Weifang, China (2017)	43
44	*S*. *abortus equi* A15	0.600	High	Donkey feces, Yantai, China (2023)	44
45	*S*. *abortus equi* A14	1.000	High	Donkey feces, Jinan, China (2023)	45
46	*S*. *abortus equi* A10	1.000	High	Donkey feces, Yantai, China (2019)	46
47	*S*. *abortus equi* A7	0.100	Medium	Donkey feces, Weifang, China (2017)	47
48	*S*. *abortus equi* A6	0.250	Medium	Donkey feces, Yantai, China (2022)	48
49	*S*. *abortus equi* A4	0.800	High	Donkey feces, Jinan, China (2017)	49
50	*S*. *abortus equi* LCS1	0.700	High	Donkey feces, Weifang, China (2022)	50
51	*S*. *abortus equi* A11	0.500	High	Donkey feces, Weifang, China (2019)	51
52	*S*. *abortus equi* A9	0.600	High	Donkey feces, Qingdao, China (2018)	52
53	*S*. *abortus equi* A3	1.000	High	Donkey feces, Yantai, China (2017)	53
54	*S*. *abortus equi* A5	1.000	High	Donkey feces, Jinan, China (2017)	54
55	*S. enteritidis* LN248	0.150	Medium	Chicken embryo, Weifang, China(2023)	55
56	*S*. *abortus equi* S2	1.000	High	Donkey feces, Qingdao, China (2016)	56
57	*S. enteritidis S*414	–	–	Chicken embryo, Yantai, China (2015)	57
58	*S. enteritidis S*415	–	–	Pig feces, Qingdao, China (2016)	58
59	*S. enteritidis S*417	0.750	High	Pig feces, Jinan, China (2016)	59
60	*S. enteritidis S*419	–	–	Pig feces, Jinan, China (2017)	60

EOP values were determined by the ratio of phage particles of 4FS1 from each susceptible Salmonella strain to the PFUs from the indicator strain of *S. abortus equi* S1. High EOP is ≥ 0.5, medium is 0.1 ≤ EOP < 0.5, low is 0.001 < EOP < 0.1, and “–” is ≤ 0.001.

### 3.5 Genomic characterization of phage 4FS1 and comparative analysis

Phage 4FS1 has a 42,485 bp double-stranded DNA with 49.07% G + C content. It contains 56 ORFs were identified, 18 in the plus strand and the rest in the minus strand, with 24 annotated as functional genes. No genes related to virulence, lysogenicity, or drug resistance were detected. The genome sequence and annotations are available in GenBank (accession number PP537413), and a detailed list of predicted ORFs is in Supplementary Table S1.

BLASTn analysis reveals that phage 4FS1 closely resembles *S. abortus equi* phage Shelby (GenBank accession number: NC_073193) with 93% query coverage and 97.87% identity. Previous research found that phage depolymerases mainly located in the tail spike or fiber. NCBI-BLASTp and HHpred analysis of ORF36 (Dpo36) reveals it a tail fiber protein with a central *β*-helix structure (125 to 676 amino acid) similarity to the depolymerase of endorhamnosidase, suggesting Dpo36 likely acts as a phage polysaccharide depolymerase.

A genome alignment reveals that phage 4FS1 is part of the *Caudoviricetes* class and *Jerseyvirus* genus. According to phylogenetic analysis of the whole genome and terminase large subunit sequence, phage 4FS1 shares a close relationship with phage Vb_SenS_SalS-S10 (NC_073193.1) and phage Vb_SenS-EnJE1 (NC_073187.1), respectively (Supplementary Figure S2).

### 3.6 Homology modeling and active site of Dpo36

The trimeric structure of Dpo36, predicted by SWISS-MODEL, consists of three monomers with N-terminal receptor binding domains separated by *α*-helices from the central and C-terminal domains, the central of which features a long β-helical region, typical of depolymerases (41) (Supplementary Figure S3).

### 3.7 Expression and antibacterial activity of Dpo36

**Figure 3:**
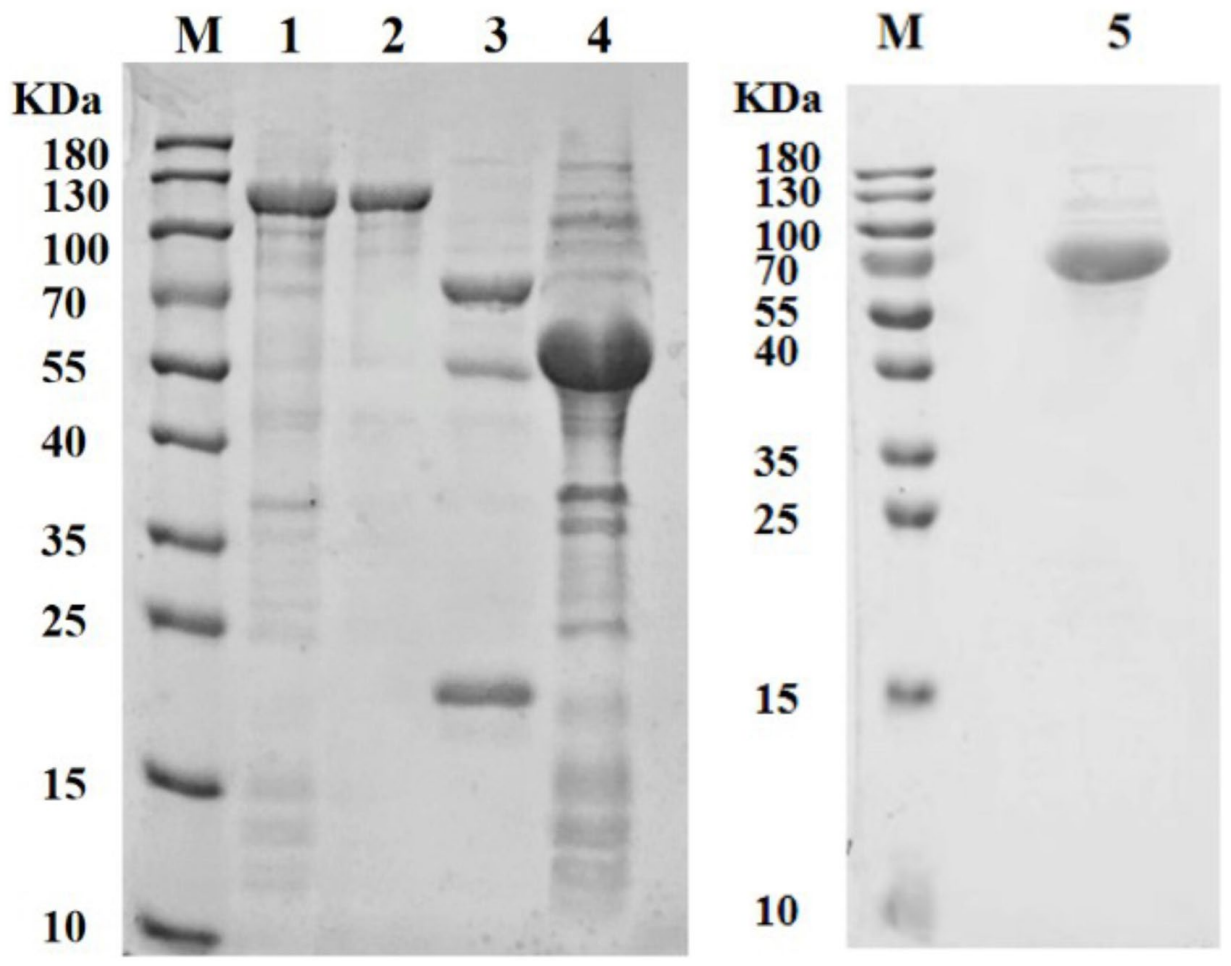
SDS-PAGE analysis of Dpo36. M represents protein standards; 1. Supernatant of pColdTF-Dpo36 expression; 2. The purified pColdTF-Dpo36 (124 KDa); 3. pColdTF-Dpo36 digested by HRV 3C Protease is composed of Dpo36 (72 KDa), pColdTF (52 KDa), and HRV 3C Protease (22 KDa); 4. Expression of pColdTF plasmid (52 KDa); 5. The purified Dpo36 (72 kDa).

The recombined Dpo36 proteins were successfully expressed in soluble form in *E. coli* BL21, and purified via affinity chromatography, yielding 1.42 mg/mL of 124 kDa pColdTF-Dpo36 (Figure 3). At concentrations of 1 mg/mL-1 μg/mL, pColdTF-Dpo36/Dpo36 demonstrated clear halos in the polysaccharide degradation spot assay. There was no significant activity difference detected between Dpo36 with or without pColdTF after H3C digestion (Supplementary Figure S4).

### 3.8 Degradation activity of polysaccharides by Dpo36

**Figure 4:**
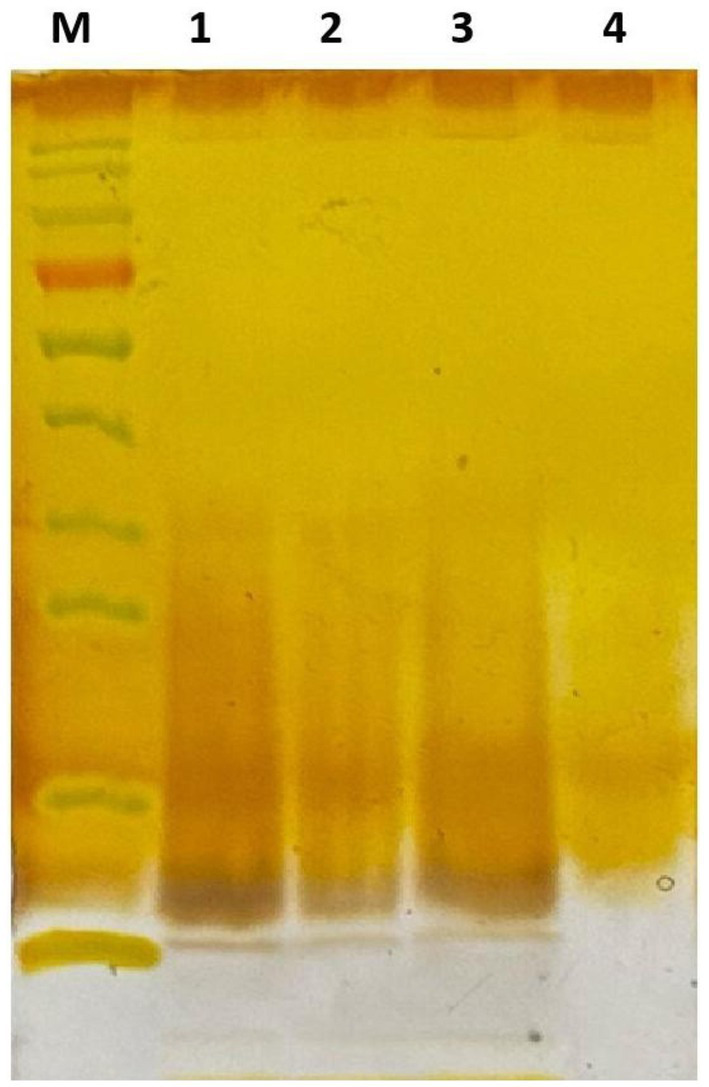
Dpo36 degradation of the polysaccharides through silver staining. M: Protein Standards; 1: polysaccharides extracts mixed with equal volume of PBS for 1 h at 37°C; 2: polysaccharides extracts mixed with equal volume of 100 μg/mL Dpo36 for 1 h at 37°C; 3: polysaccharides extracts mixed with equal volume of 100 μg/mL inactivated Dpo36 for 1 h at 37°C; 4: Dpo36 mixed with equal volume of PBS for 1 h at 37°C.

**Figure 5:**
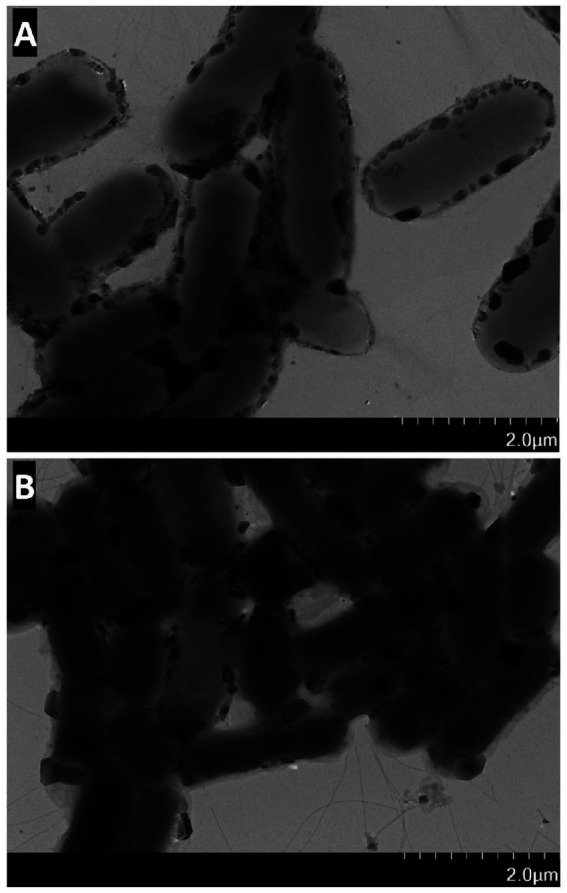
The effect of *S. abortus equi* S1 by Dpo36. **(A)** TEM image of *S. abortus equi* S1 treated with Dpo36. **(B)** TEM image of *S. abortus equi* S1 treated without Dpo36.

Polysaccharide extracts treated with Dpo36 exhibited a decreased intensity versus controls. The thermal inactivation of Dpo36 prevented the degradation of polysaccharides, as illustrated in Figure 4, suggesting its involvement in this process. TEM revealed that treatment with Dpo36 result in smoother bacterial surfaces and reduced aggregation, as shown in Figure 5.

### 3.9 Environmental stability of Dpo36

**Figure 6:**
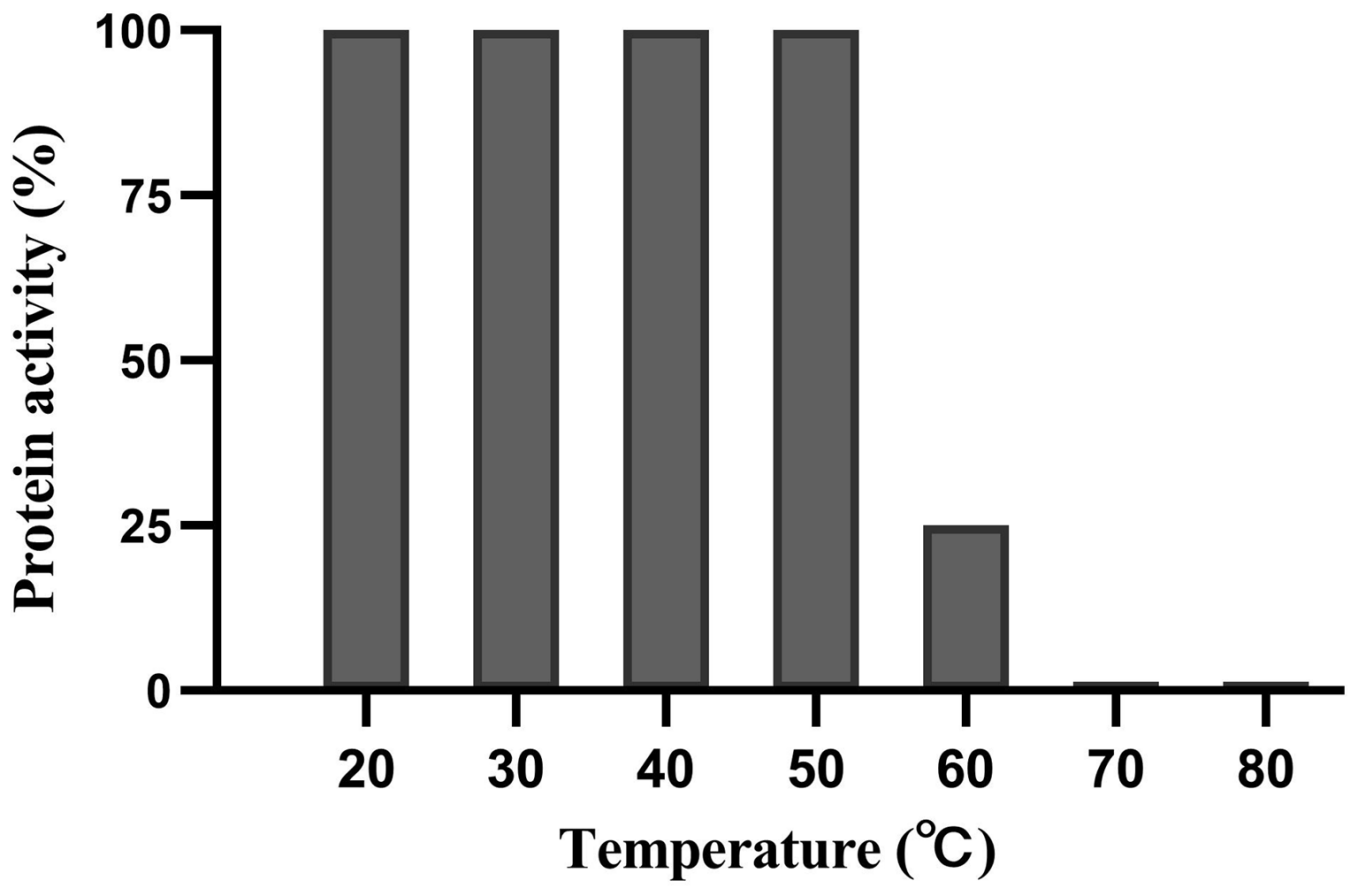
Thermal stability of Dpo36. Effects of Dpo36 on the values of *S. abortus equi* S1 at temperatures ranging from 20 to 80°C.

**Figure 7:**
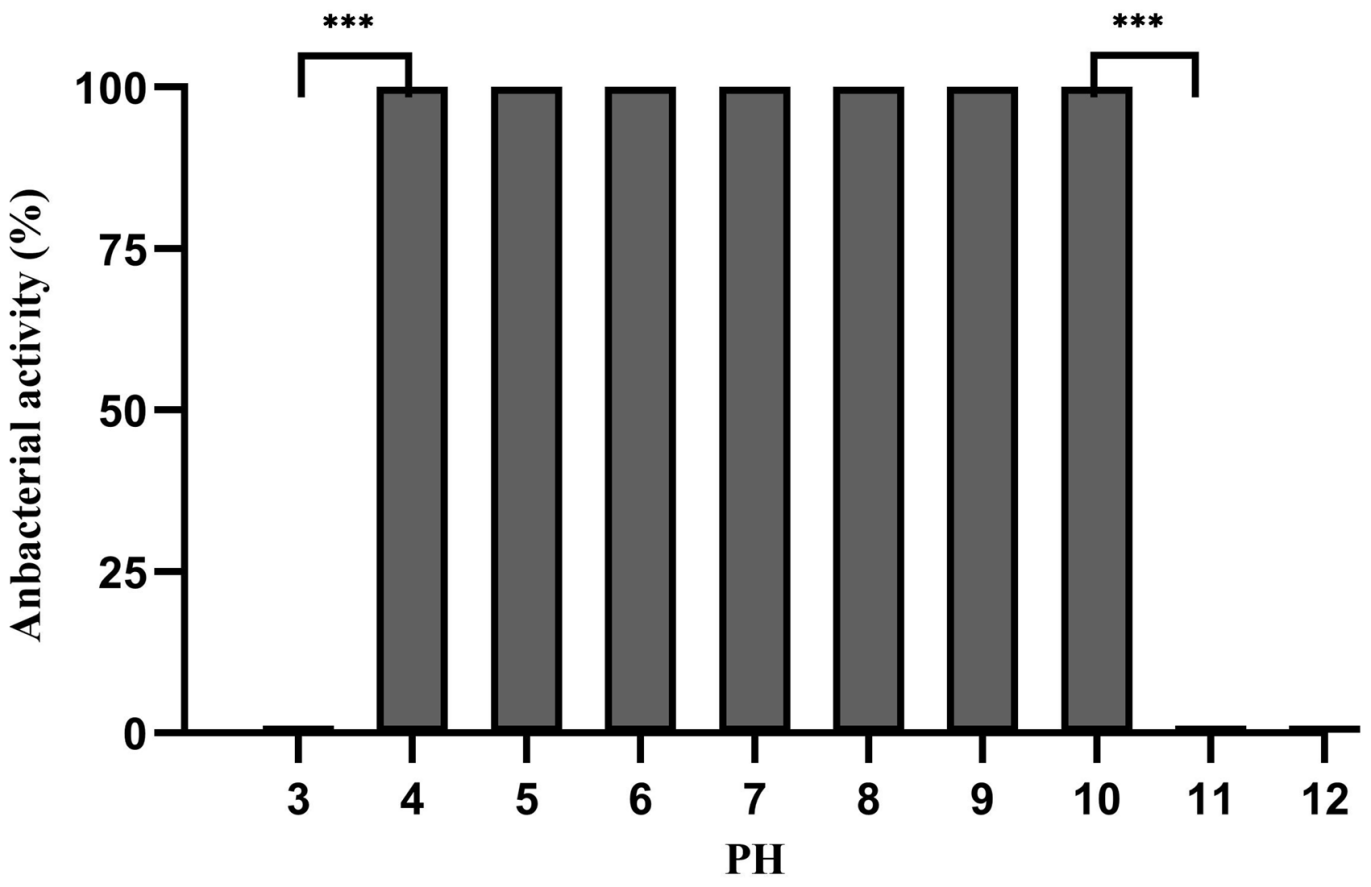
pH stability of Dpo36. Effects of Dpo36 on the values of *S. abortus equi* S1 at pH ranging from 3 to 12.

Dpo36 remained stable below 50°C, but was completely inactivated at 70°C after 60 min (Figure 6). Dpo36 remained stable at a pH ranging from 4 to 10 after 60 min, and completely inactivated at pH 3/11 after 60 min (Figure 7).

### 3.10 Biofilm degradation activity of Dpo36

**Figure 8:**
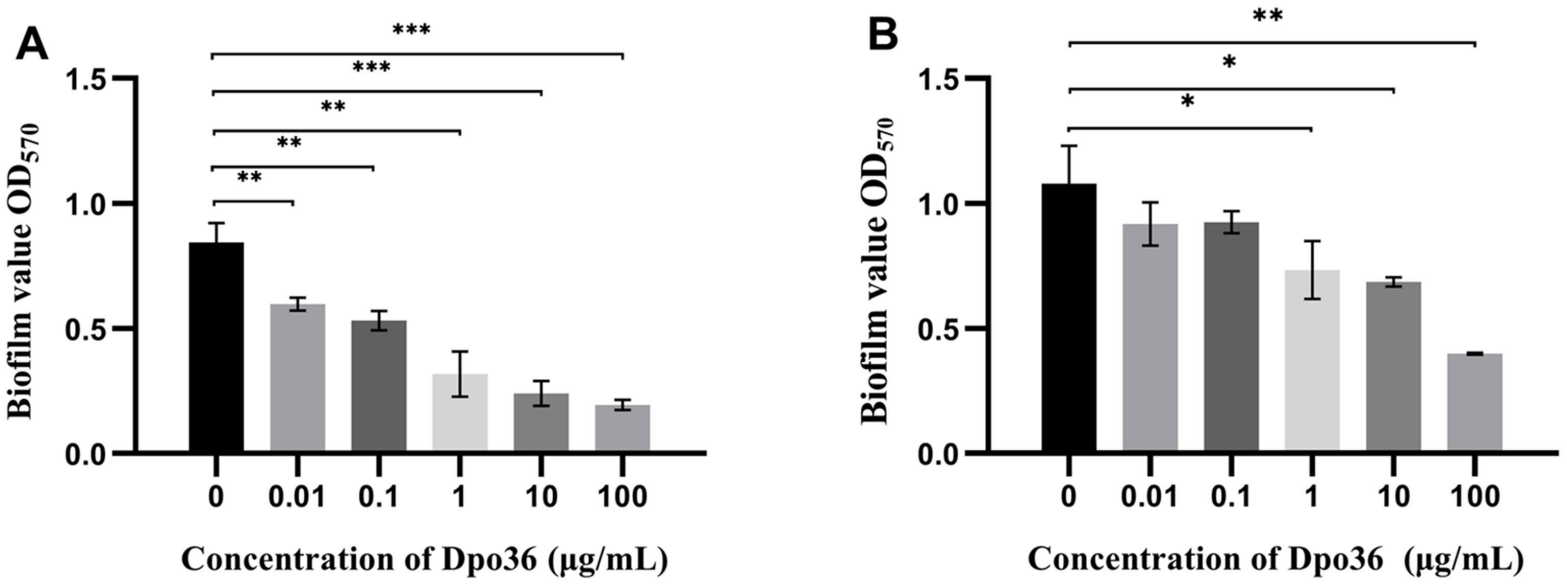
Anitibiofilm activity of Dpo36. **(A)** Different concentrations of Dpo36 inhibited biofilm production. **(B)** The formed biofilm was degraded under different concentrations of Dpo36. *: *p* < 0.05; **: *p* < 0.01; ***: *p* < 0.001.

The efficacy of Dpo36 in inhibiting and degrading biofilms was assessed at various concentrations. It significantly inhibited biofilm formation and degraded preformed biofilms at 100, 10, 1, 0.1, and 0.01 μg/mL after 2 h (Figures 8A,B).

### 3.11 The inhibitory effect of Dpo36 combined with donkey serum on bacteria *in vitro*

**Figure 9:**
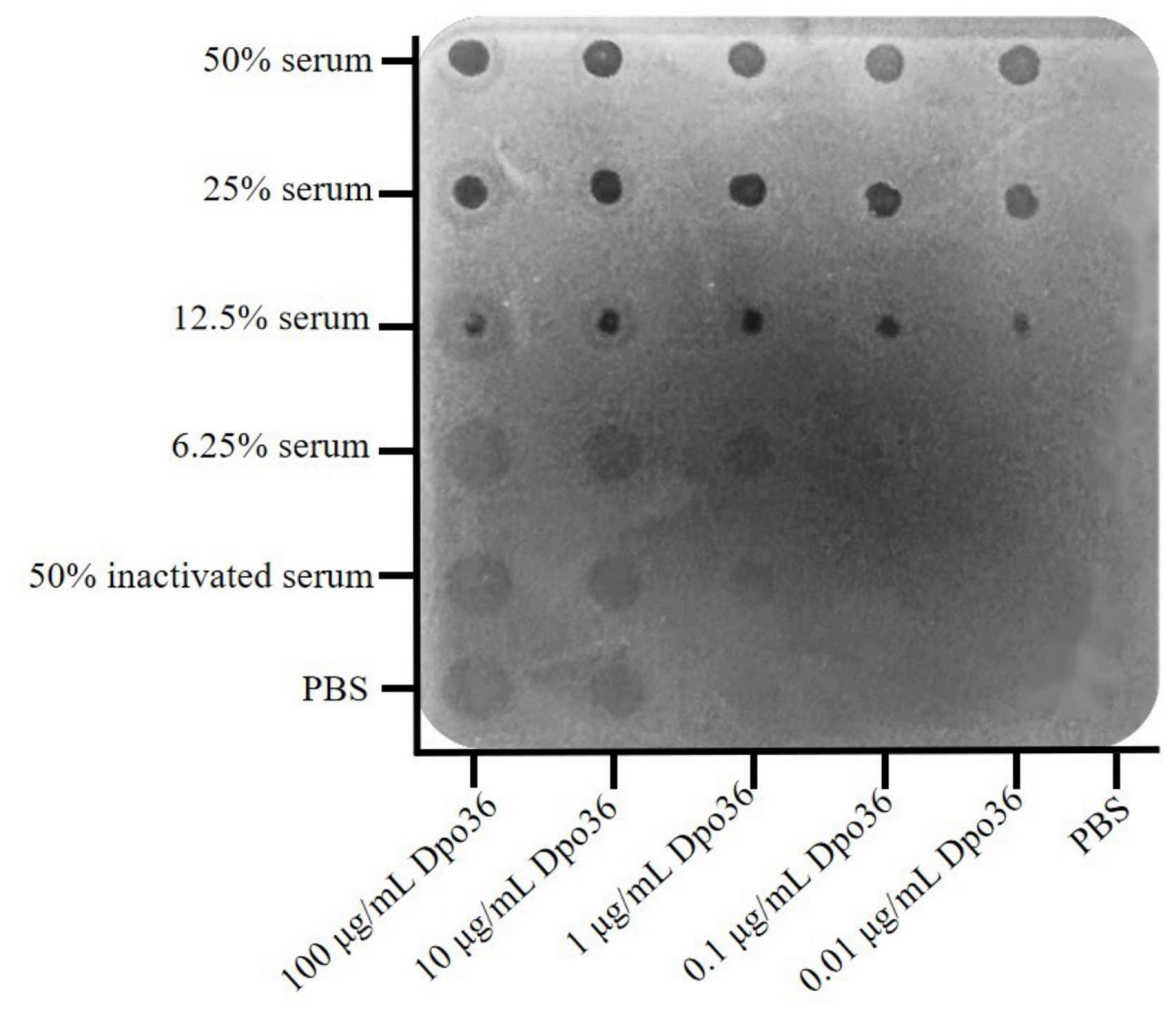
The different concentrations of Dpo36 bacteriostatic activity in the presence of serum from healthy donkeys.

The spot assay demonstrated that the combination of Dpo36 with donkey serum significantly inhibited bacterial growth, even at low ratio of serum (12.5%) with Dpo36 from 0.01 to 100 μg/mL, resulting in distinct inhibitory zones (Figure 9). Conversely, neither serum alone nor inactivated serum combined with Dpo36 exhibited any bacteriostatic effect, and Dpo36 alone produced only a translucent halo.

Furthermore, treatment with varying concentrations of Dpo36 and donkey serum significantly decreased the OD_600_ of *S. abortus equi* S1 after 24 h at 37°C. In the group with 25% serum, Dpo36 at 1, 10, and 100 μg/mL significantly inhibited bacterial growth in the first 8 h, but this effect decreased over time. After 24 h, the bacteriostatic effect of 1 μg/mL of Dpo36 with 25% serum was less effective than with 12.5% serum (Supplementary Figures S5A,B). The combination of 25% serum and 100 μg/mL Dpo36 reduced the OD_600_ value by about 0.3 compared to the control (*p* < 0.05) (Supplementary Figure S5B). Similarly, 12.5% serum with 100 μg/mL Dpo36 reduced the OD_600_ by about 0.2 compared to the control (*p* < 0.05) (Supplementary Figure S5B). Additionally, 10 μg/mL Dpo36 with either 12.5% or 25% donkey serum significantly inhibited bacterial growth (*p* < 0.05) (Supplementary Figures S5A,B), whereas neither Dpo36 nor serum alone, nor their combination with inactivated serum, demonstrated any bacteriostatic effects.

### 3.12 Prevention and treatment of Dpo36 in mice infected by *S. abortus equi*

**Figure 10:**
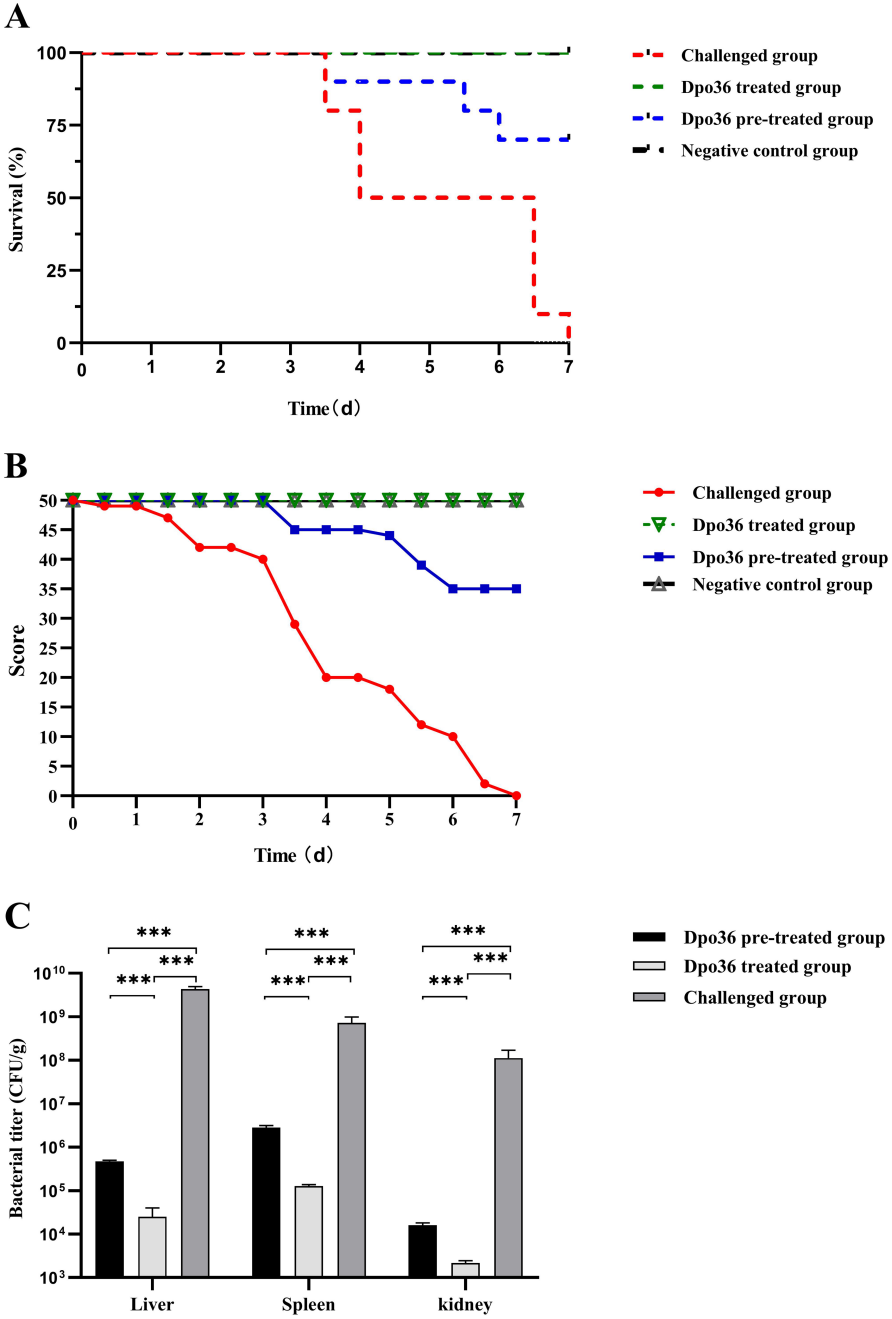
Effects of Dpo36 on the survival of mice challenged with *S. abortus equi* S1. **(A)** Changes in *in vivo* survival rates of *S. abortus equi* S1-challenged mice after treatment with Dpo36. **(B)** Changes in *in vivo* body condition score test results of *S. abortus equi* S1-challenged mice after treatment with Dpo36. **(C)** Changes in the number of *S. abortus equi* S1 in viscera of mice after treatment with Dpo36. *: *p* < 0.05; **: *p* < 0.01; ***: *p* < 0.001.

Both the pre-treated and treated groups had significantly higher survival rates and posture scores than those of the control group, with the treated group outer performing the pre-treated group (Figures 10A,B). On day 7 post-infection, all mice were euthanized, and *S. abortus equi* S1 was quantified from mouse tissues (Figure 10C). Our findings indicated that the treated group had the lowest bacterial load in tissues and no mortality, with body condition scores similar to the negative control group. The pre-treated group had a higher bacterial load (10^4^–10^6^ CFU/g) than the treated group (10^3^–10^5^ CFU/g), but much lower than the challenged group (>10^8^ CFU/g) (Figure 10C). These findings suggest that administering Dpo36 after a challenge is the most effective strategy, as it significantly reduces bacterial load in various organs and enhances survival rate in mice.

## 4 Discussion

*Salmonella abortus equi* infection poses a significant challenge in donkey breeding, resulting in decreased reproductive efficiency and foal survival rates (42, 43). This necessitates the development of novel strategies to address multidrug-resistant (MDR) bacteria while minimizing the risk of resistance development (44).

Phages have emerged as promising antimicrobial agents (45, 46), demonstrating substantial therapeutic potential by selectively targeting and eradicating pathogens, while preserving the commensal microbiota (47). This selective bactericidal activity has facilitated the effective treatment and prevention of bacterial infections (17, 48).

Phage 4FS1, as determined by EOP assays, exhibited a broad host range against *Salmonella* species and achieved high titers (~10^8^ PFU/mL) across most host strains. Its rapid growth dynamics suggest its potential for effective biocontrol of the pathogen. Notably, phage 4FS1 exhibited a shorter latency period and a larger burst size in the *S. abortus equi* S1 host strain compared to other *Salmonella* phages (49, 50), indicating enhanced pathogen-killing efficacy. In terms of environmental stability, phage 4FS1 demonstrates optimal efficacy at a neutral pH range of 6 to 8, with a marked decline in activity outside the pH range of 5 to 10. It exhibits stability at temperatures between 37°C and 50°C for 1 h and retains significant activity following 1 h of UV exposure, rendering it suitable for application in environmental disinfection. In the context of donkey husbandry, salt intake is typically achieved through the licking of salt blocks. To assess the potential for phage stability with salt blocks, commonly used in donkey husbandry, salt tolerance assays were conducted. The study demonstrated that phage 4FS1 maintained stability in high-salt environments for 4 h. Consequently, phage 4FS1 exhibits potential as a therapeutic agent for bacterial infections in the donkey intestine, potentially administered through the licking of salt bricks. Furthermore, genomic analysis elucidated protein functions and verified the safety of phage 4FS1 for practical applications.

Although phages are effective in eradicating bacteria, they can also contribute to the emergence of resistant bacterial strains (51). Conversely, depolymerases do not directly induce bacterial cell death, they only exert a minimal influence on resistance development (52). The growth of the phage 4FS1 spot over time manifests as a translucent halo, indicating depolymerase production. Genomic analysis indicates that ORF36 is linked to the gene for the tail spike protein, suggesting a role in phage depolymerase activity. Structural modeling predicts that ORF36 has an elongated, right-handed *β*-helical structure (Supplementary Figure S3), aiding in polysaccharide degradation and phage penetration. It also enhances the complementary receptor recognition on the host surface and boosts immune responses to eliminate bacteria (53). Dpo36, a large, acidic, hydrophilic protein lacking signal peptides or transmembrane domains, was characterized as the phage-encoded depolymerase. This enzyme possesses a modular architecture comprising of three distinct domains and demonstrates stability under extreme conditions, including temperatures up to 50°C and pH ranging from 4 to 10. While Dpo36 does not inherently possess bactericidal properties, it exhibits *in vitro* activity and potential utility in the degradation of polysaccharides, maintaining its functionality at elevated temperatures. Experimental findings suggest that Dpo36 is capable of preventing biofilm formation by degrading polysaccharides and enhancing immune responses, such as increasing serum complement deposition, which significantly improves antibacterial efficacy, aligning with previous research (14, 54). *In vitro* studies demonstrated that Dpo36, when combined with donkey serum, exhibits a substantial bactericidal effect without compromising its activity. This effect is likely attributable to the removal of the bacterial surface barrier, thereby facilitating complement system activity (14). Interestingly, the antibacterial effect of 1 μg/mL Dpo36 in combination with 12.5% serum exceeded that observed with 25% serum at the 24-h mark (Supplementary Figure S5). This observation indicates that while the bacteriostatic effect of Dpo36 is positively correlated with its concentration when serum concentration is held constant, the nutrients present in the serum may interfere with accurately assessing the bacteriostatic efficacy of Dpo36. Preliminary data suggest that mouse serum exhibits a weaker synergistic effect compared to donkey serum (data not shown). The underlying mechanisms responsible for this differential efficacy warrant further investigation.

Following the validation of the therapeutic efficacy of Dpo36 *in vitro*, it is imperative to establish its activity *in vivo.* Consequently, its effectiveness was evaluated through animal experiments. The results indicated a significant improvement in survival rates and body condition scores in both treated and pre-treated mice compared to the challenged group, along with a reduction in bacterial levels. These outcomes support its potential in infection management and align with findings from previous studies (39, 55). This is the first report on phage depolymerase against *S. abortus equi*, and further research is needed to evaluate the potential of Dpo36 in preventing and managing *S. abortus equi* infection in donkeys.

## 5 Conclusion

This study identified and characterized a lytic phage, 4FS1, and its depolymerase. Phage 4FS1 demonstrated significant environmental stability, a broad host range against *Salmonella* species, high titers, a short latency period, and a substantial burst size. Furthermore, the phage-derived depolymerase, Dpo36, was identified and shown to effectively disrupt biofilms and exhibit potent antimicrobial activity against *S. abortus equi* in both *in vitro* and *in vivo* settings. These findings suggest that Dpo36 holds potential as a novel therapeutic agent for the treatment of bacterial infections.

## Data Availability

The original contributions presented in the study are included in the article/Supplementary material, further inquiries can be directed to the corresponding author.

## Footnotes

^1^ http://rast.nmpdr.org

^2^ http://opal.biology.gatech.edu/Gene.Mark/

^3^ http://trna.ucsc.edu/tRNAscan-SE/

^4^ http://phaster.ca/

^5^ https://cge.cbs.dtu.dk/services/MLST/

^6^ https://swissmodel.expasy.org/

^7^ https://www.ncbi.nlm.nih.gov/Structure/cdd/wrpsb.cgi

## Supplementary material

SUPPLEMENTARY FIGURE S1 Environmental stability of phage 4FS1. (A) Thermal stability of phage 4FS1: The phage titers showed no significant differences at 37°C–50°C. (B) fpH stability of phage 4FS1: Phage 4FS1 was stable over the range of pH (5–10). (C) UV stability of phage 4FS1: The phage titers still maintain 10^7^ PFU/mL at UV for 1 hour. (D) Salt stability of phage 4FS1: The phage titers showed no significant difference at 2.5%-15.0% NaCl. Data are presented as mean ± SD (*n* = 3).

SUPPLEMENTARY FIGURE S2 Phylogenetic trees show the relationship between phage 4FS1 and other phages in the NCBI database. (A) A phylogenetic tree based on the whole genome. (B) A phylogenetic tree based on the terminase large subunit.

SUPPLEMENTARY FIGURE S3 3D model of Dpo36 predicted by SWISS-MODEL. The modular structure of Dpo36 consists of the N-terminal domain, central domain, and C-terminal domain. (A) Monomer model of Dpo36; (B) Trimeric model of Dpo36.

SUPPLEMENTARY FIGURE S4 pColdTF-Dpo36/Dpo36 degradation activity measured using the spot assay at different concentrations. 1: Dpo36 (1 mg/mL); 2: Dpo36 (100 μg/mL); 3: Dpo36 (10 μg/mL); 4: Dpo36 (1 μg/mL); 5: Dpo36 (0.1 μg/mL).

SUPPLEMENTARY FIGURE S5 The inhibitory effect of Dpo36 combined with serum against *S. abortus equi* S1. (A) The inhibitory effect on bacterial growth was assessed using 25% serum and Dpo36 at concentration of 100, 10, and 1 μg/mL. (B) The inhibitory effect on bacterial growth was assessed using 12.5% serum and Dpo36 at concentration of 100, 10, 1 μg/mL of Dpo36. The combination of Dpo36 with either 12.5% or 25% serum significantly inhibited bacterial proliferation. In contrast, neither serum alone, Dpo36 alone, nor inactivated serum combined with Dpo36 exhibited any inhibitory effect. Positive control: Bacterial with no serum and Dpo36; Negative control: LB broth.
