# Supplementary figures and images for: Characterization of a Salmonella abortus equi phage 4FS1 and its depolymerase
Source: Front Vet Sci. 2024 Nov 25;11:1496684. doi: 10.3389/fvets.2024.1496684 (PMC11626802; doi:10.3389/fvets.2024.1496684)

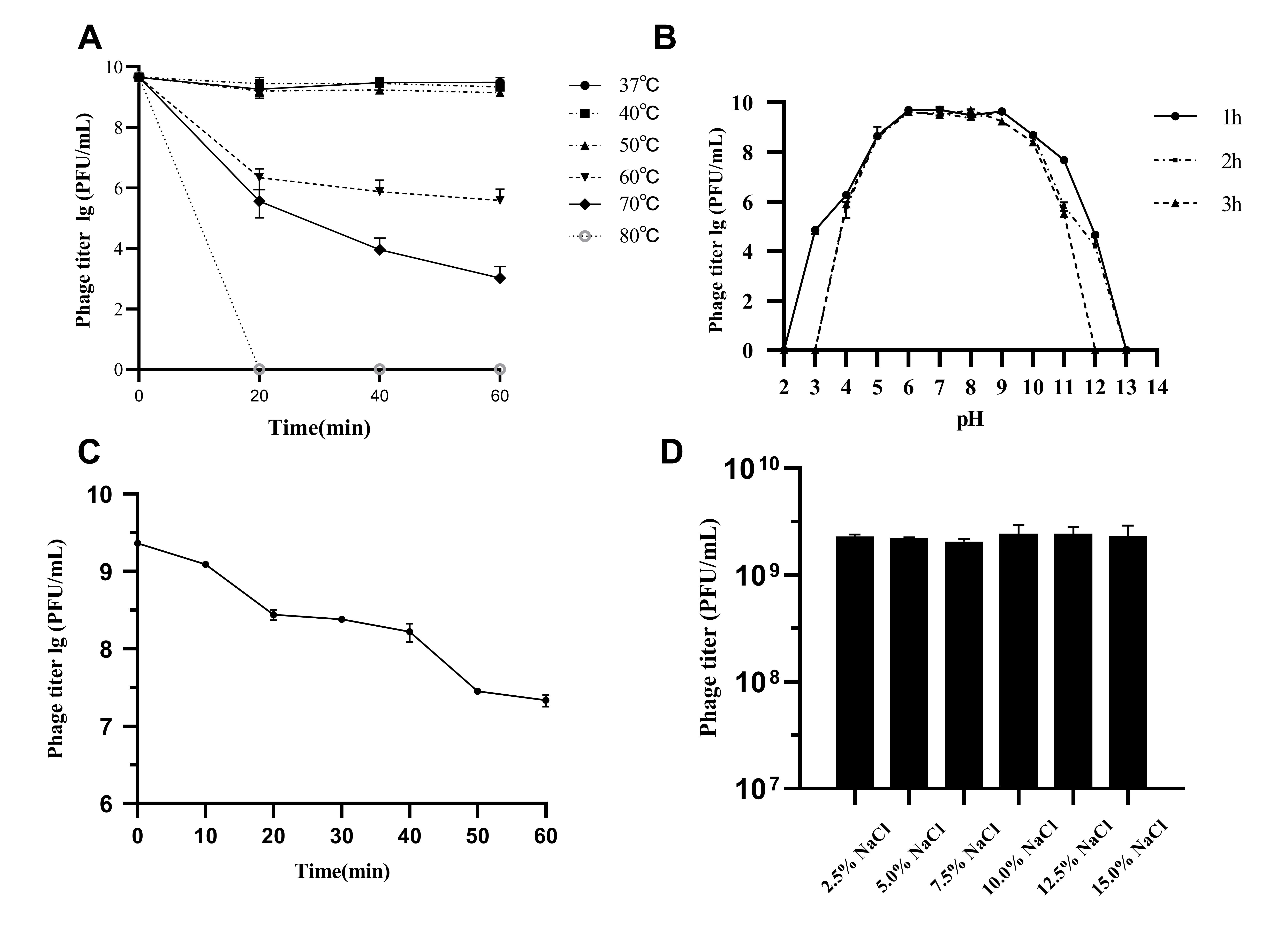

Supplement: SUPPLEMENTARY FIGURE S1 — Environmental stability of phage 4FS1. (A) Thermal stability of phage 4FS1: The phage titers showed no significant differences at 37°C–50°C. (B) fpH stability of phage 4FS1: Phage 4FS1 was stable over the range of pH (5–10). (C) UV stability of phage 4FS1: The phage titers still maintain 107 PFU/mL at UV for 1 hour. (D) Salt stability of phage 4FS1: The phage titers showed no significant difference at 2.5%-15.0% NaCl. Data are presented as mean ± SD (n = 3). [file Image_1.jpg]

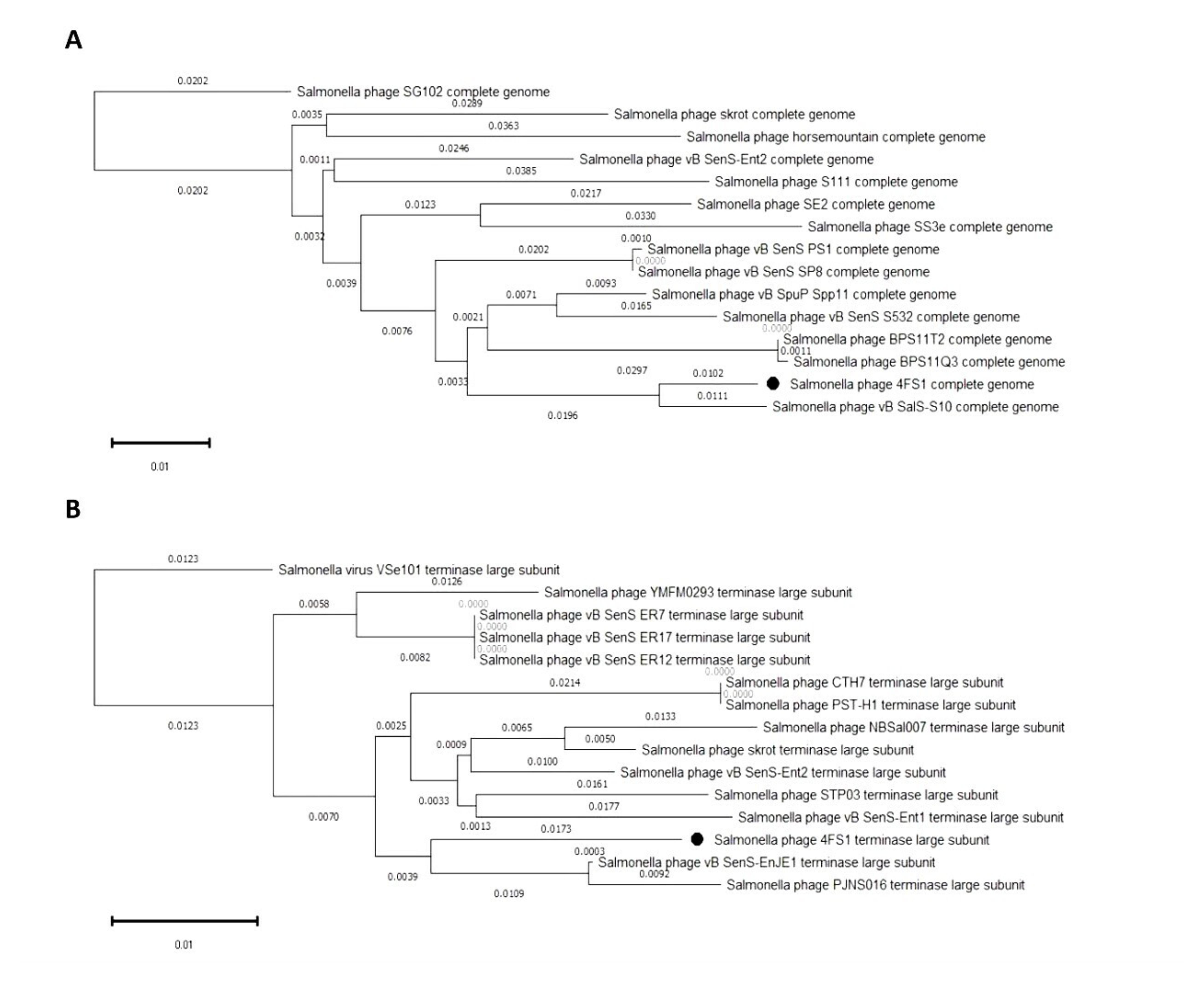

Supplement: SUPPLEMENTARY FIGURE S2 — Phylogenetic trees show the relationship between phage 4FS1 and other phages in the NCBI database. (A) A phylogenetic tree based on the whole genome. (B) A phylogenetic tree based on the terminase large subunit. [file Image_2.JPEG]

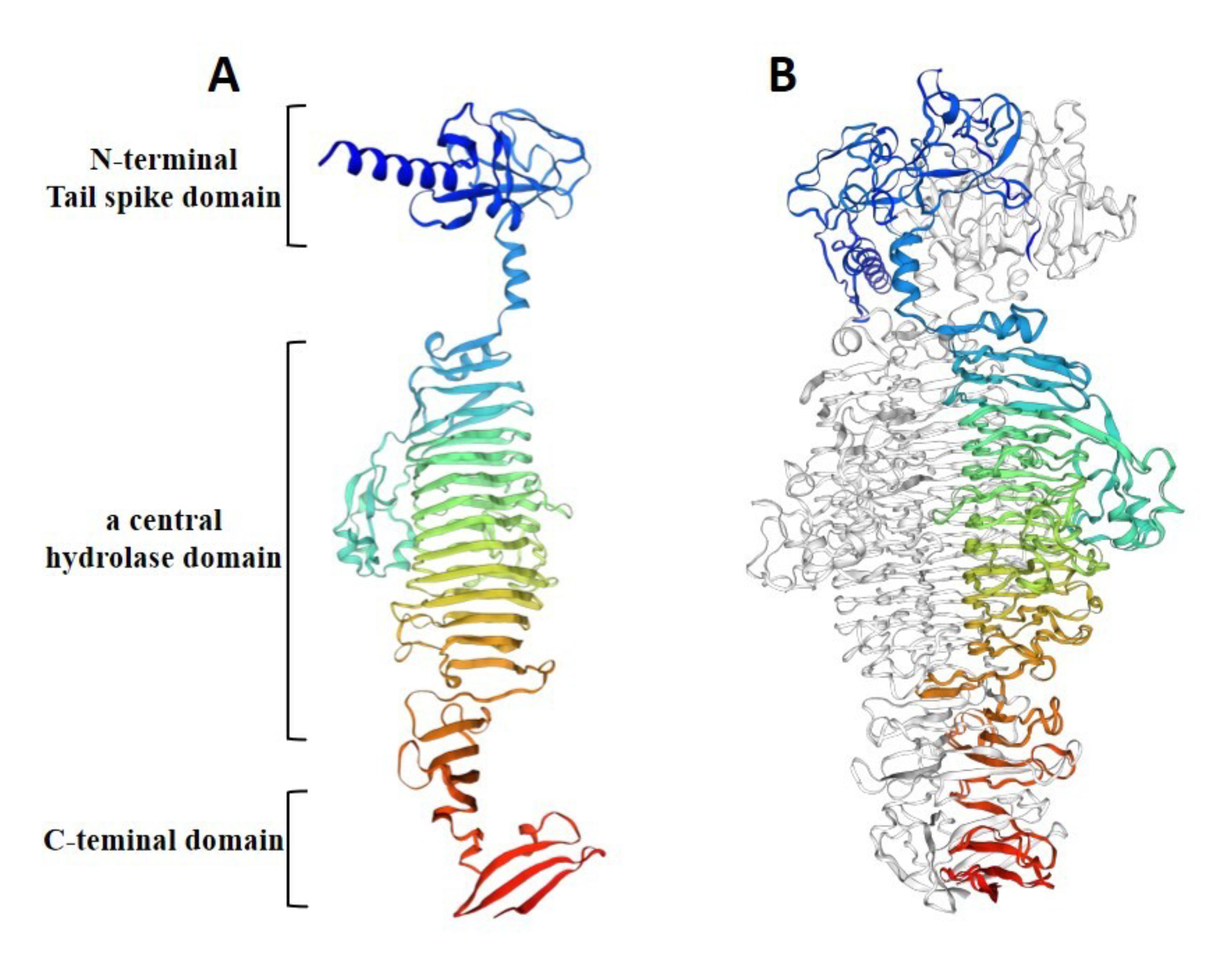

Supplement: SUPPLEMENTARY FIGURE S3 — 3D model of Dpo36 predicted by SWISS-MODEL. The modular structure of Dpo36 consists of the N-terminal domain, central domain, and C-terminal domain. (A) Monomer model of Dpo36; (B) Trimeric model of Dpo36. [file Image_3.JPEG]

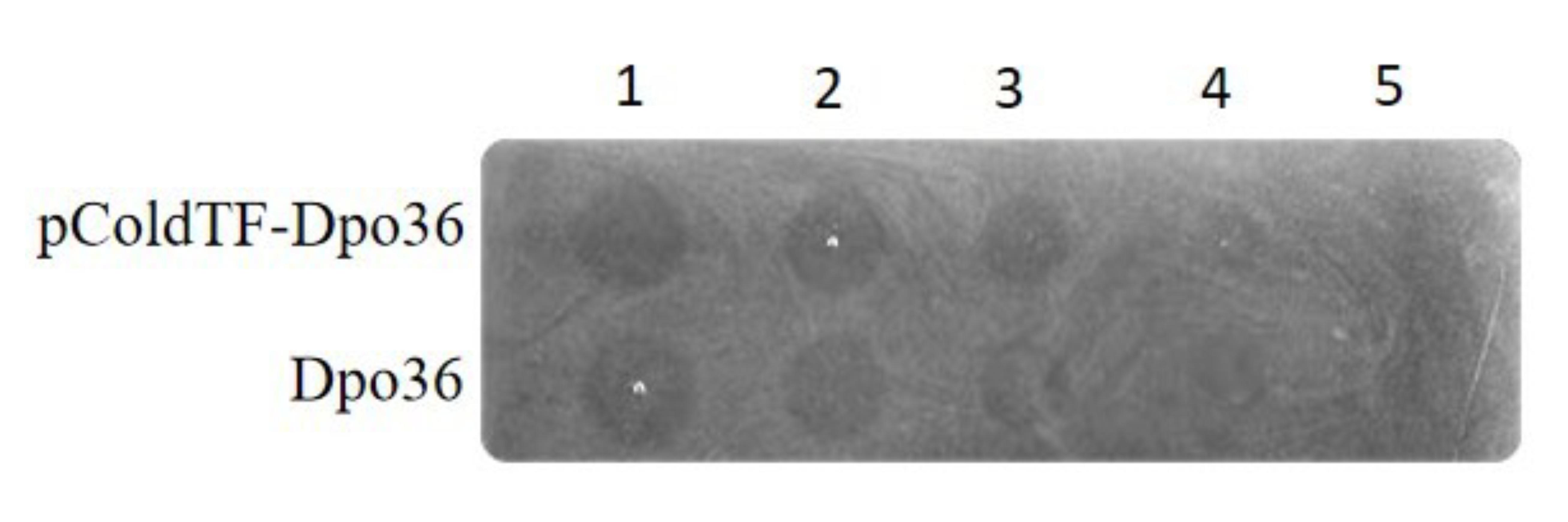

Supplement: SUPPLEMENTARY FIGURE S4 — pColdTF-Dpo36/Dpo36 degradation activity measured using the spot assay at different concentrations. 1: Dpo36 (1 mg/mL); 2: Dpo36 (100 μg/mL); 3: Dpo36 (10 μg/mL); 4: Dpo36 (1 μg/mL); 5: Dpo36 (0.1 μg/mL). [file Image_4.JPEG]

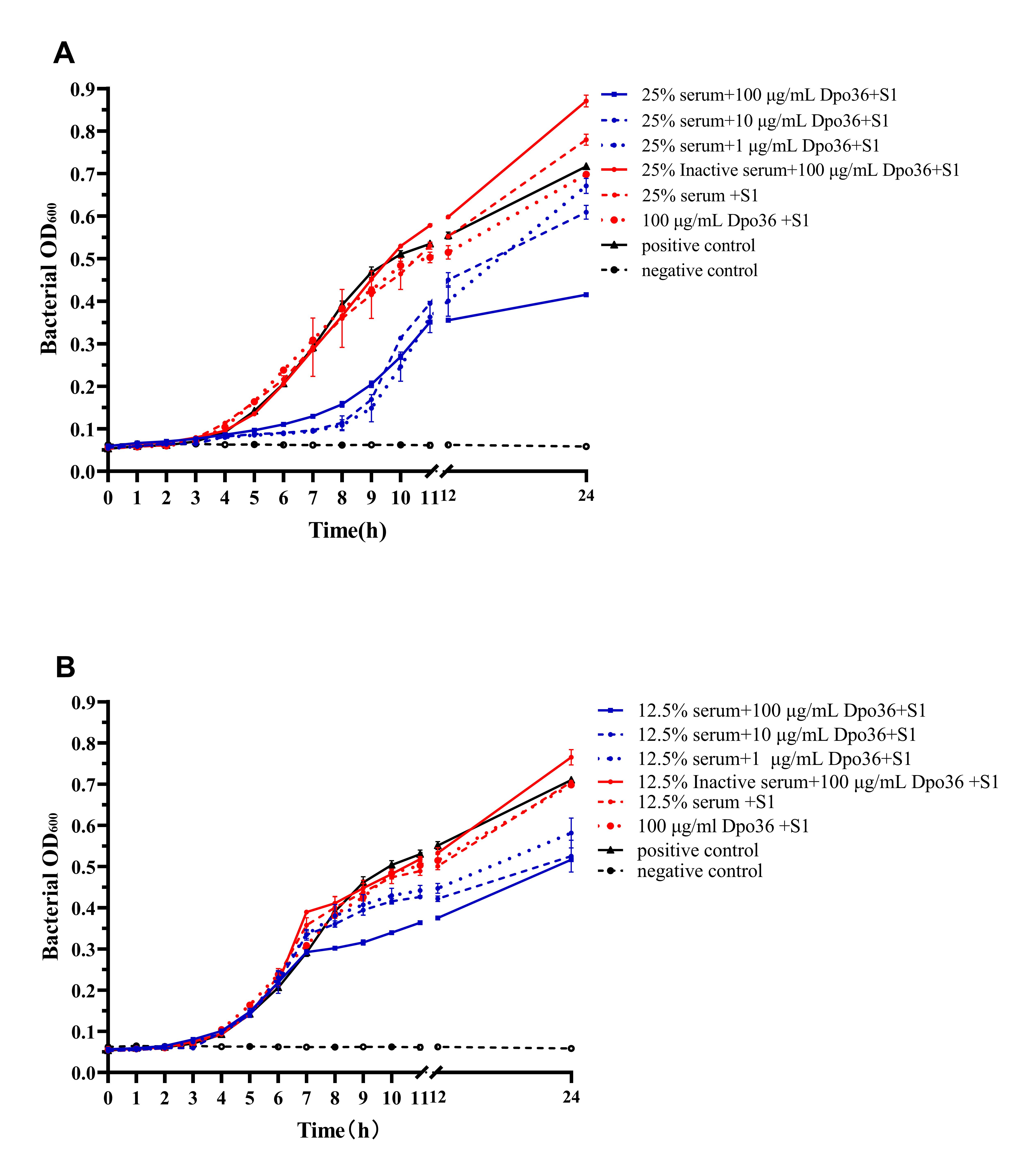

Supplement: SUPPLEMENTARY FIGURE S5 — The inhibitory effect of Dpo36 combined with serum against S. abortus equi S1. (A) The inhibitory effect on bacterial growth was assessed using 25% serum and Dpo36 at concentration of 100, 10, and 1 μg/mL. (B) The inhibitory effect on bacterial growth was assessed using 12.5% serum and Dpo36 at concentration of 100, 10, 1 μg/mL of Dpo36. The combination of Dpo36 with either 12.5% or 25% serum significantly inhibited bacterial proliferation. In contrast, neither serum alone, Dpo36 alone, nor inactivated serum combined with Dpo36 exhibited any inhibitory effect. Positive control: Bacterial with no serum and Dpo36; Negative control: LB broth. [file Image_5.JPEG]
