# Supplementary material for: Characterization of a Salmonella abortus equi phage 4FS1 and its depolymerase
Source: Front Vet Sci. 2024 Nov 25;11:1496684. doi: 10.3389/fvets.2024.1496684 (PMC11626802; doi:10.3389/fvets.2024.1496684)
Supplement: Supplementary file 6 [file Table_1.DOCX]

**Table S1.** Predicted ORFs in phage 4FS1 and their annotation.

| **ORFs** | **Start** | **Stop** | **Strand** | **Function** | **[Per.Ident](https://blast.ncbi.nlm.nih.gov/Blast.cgi?CMD=Get&ADV_VIEW=yes&ADV_VIEW=on&ALIGNMENTS=100&ALIGNMENT_VIEW=Pairwise&CDD_RID=5X0T8V3V013&CDD_SEARCH_STATE=1&CONFIG_DESCR=Ds,ClustMemNbr,ClustTaxNbr,ClustComn,Sc,Ms,Ts,Cov,Eval,Idnt,AccLen,Acc&DATABASE_SORT=0&DESCRIPTIONS=100&DYNAMIC_FORMAT=on&FIRST_QUERY_NUM=0&FORMAT_NUM_ORG=1&FORMAT_OBJECT=Alignment&FORMAT_PAGE_TARGET=&FORMAT_TYPE=HTML&GET_SEQUENCE=yes&I_THRESH=&LINE_LENGTH=60&MASK_CHAR=2&MASK_COLOR=1&NEW_VIEW=yes&NUM_OVERVIEW=100&PAGE=Proteins&QUERY_INDEX=0&QUERY_NUMBER=0&RESULTS_PAGE_TARGET=&RID=5X0T8RNC016&SHOW_LINKOUT=yes&SHOW_OVERVIEW=yes&SORT_BY=3&STEP_NUMBER=&WORD_SIZE=6&ADV_VIEW=on&DISPLAY_SORT=3&HSP_SORT=3)(%)** | **Accession** |
| --- | --- | --- | --- | --- | --- | --- |
| ORF1 | 908 | 1609 | 701 | scaffold protein | 99.6 | YP_009191586.1 |
| ORF2 | 1803 | 2189 | 386 | hypothetical protein | 92.2 | YP_009035144.1 |
| ORF3 | 2508 | 2966 | 458 | neck whiskers protein | 98 | YP_009280129.1 |
| ORF4 | 2969 | 4042 | 1073 | minor capsid protein | 97.7 | YP_009009929.1 |
| ORF5 | 4828 | 4151 | 677 | hypothetical protein | 97.7 | YP_009191590.1 |
| ORF6 | 4859 | 6331 | 1472 | DUF4055 domain-containing protein | 99.2 | YP_009035140.1 |
| ORF7 | 6344 | 7615 | 1271 | terminase family protein | 99.3 | YP_008767173.1 |
| ORF8 | 7605 | 8111 | 506 | putative terminase small subunit | 99.4 | YP_001110829.1 |
| ORF9 | 8244 | 8474 | 230 | hypothetical protein | 100 | YP_008767068.1 |
| ORF10 | 8488 | 8787 | 299 | hypothetical protein | 94.9 | YP_009009979.1 |
| ORF11 | 8787 | 9011 | 224 | NinH family protein | 100 | YP_009280138.1 |
| ORF12 | 9154 | 9333 | 179 | putative NinZ-like protein | 91.5 | YP_001110826.1 |
| ORF13 | 9330 | 9485 | 155 | DUF2737 family protein | 100 | YP_007010508.1 |
| ORF14 | 9482 | 9667 | 185 | hypothetical protein | 100 | YP_005098119.1 |
| ORF15 | 9851 | 10339 | 488 | lysozyme | 95.1 | YP_005098118.1 |
| ORF16 | 10317 | 10607 | 290 | putative class I holin | 94.8 | YP_008767059.1 |
| ORF17 | 10609 | 10890 | 281 | hypothetical protein | 100 | YP_009191600.1 |
| ORF18 | 10969 | 11334 | 365 | hypothetical protein | 96.7 | YP_009608639.1 |
| ORF19 | 11337 | 11540 | 203 | hypothetical protein | 95.5 | YP_001110819.1 |
| ORF20 | 11537 | 11875 | 338 | hypothetical protein | 91.4 | YP_005098113.1 |
| ORF21 | 12045 | 12209 | 164 | hypothetical protein | 87 | YP_009608643.1 |
| ORF22 | 13373 | 13188 | 185 | hypothetical protein | 100 | YP_224054.2 |
| ORF23 | 13603 | 13370 | 233 | hypothetical protein | 92.2 | YP_008859653.1 |
| ORF24 | 15846 | 13660 | 2186 | AAA family ATPase | 98.8 | YP_005098109.1 |
| ORF25 | 15861 | 16079 | 218 | helix-turn-helix domain-containing protein | 100 | YP_009280153.1 |
| ORF26 | 16725 | 16213 | 512 | hypothetical protein | 97.1 | YP_005098107.1 |
| ORF27 | 18008 | 16767 | 1241 | DUF2800 domain-containing protein | 92.3 | YP_009620123.1 |
| ORF28 | 18178 | 18005 | 173 | hypothetical protein | 98.2 | YP_009821873.1 |
| ORF29 | 18894 | 18268 | 626 | DUF2815 family protein | 99.5 | YP_001110811.1 |
| ORF30 | 22050 | 18952 | 3098 | intein-containing DNA polymerase precursor | 98.6 | YP_009322845.1 |
| ORF31 | 22501 | 22040 | 461 | VRR-NUC domain-containing protein | 94.7 | YP_009191561.1 |
| ORF32 | 22724 | 22533 | 191 | hypothetical protein | 96.8 | YP_009009959.1 |
| ORF33 | 23223 | 22726 | 497 | HNH endonuclease | 97 | YP_005098098.1 |
| ORF34 | 25685 | 23220 | 2465 | helicase | 98.5 | YP_009010936.1 |
| ORF35 | 25903 | 25682 | 221 | hypothetical protein | 100 | YP_009322851.1 |
| ORF36 | 26022 | 28052 | 2030 | Tail spike protein | 96.3 | YP_224071.2 |
| ORF37 | 28089 | 30575 | 2486 | tail fiber protein | 98.8 | YP_009191567.1 |
| ORF38 | 30638 | 31003 | 365 | hypothetical protein | 99.2 | YP_009322854.1 |
| ORF39 | 31000 | 31515 | 515 | DUF1833 family protein | 100 | YP_009322855.1 |
| ORF40 | 31512 | 32012 | 500 | hypothetical protein | 97.6 | YP_009608663.1 |
| ORF41 | 32014 | 34347 | 2333 | tape measure protein | 99.2 | YP_009608664.1 |
| ORF42 | 34340 | 34699 | 359 | hypothetical protein | 98.3 | YP_009280170.1 |
| ORF43 | 34705 | 35121 | 416 | hypothetical protein | 100 | YP_005098150.1 |
| ORF44 | 35470 | 35291 | 179 | superinfection immunity protein | 100 | YP_009322860.1 |
| ORF45 | 36687 | 35533 | 1154 | putative serine/threonine protein phosphatase | 99.7 | YP_009035160.1 |
| ORF46 | 36987 | 36757 | 230 | hypothetical protein | 100 | YP_009280174.1 |
| ORF47 | 37774 | 37103 | 671 | Rha family transcriptional regulator | 97.8 | YP_007010473.1 |
| ORF48 | 37806 | 38975 | 1169 | putative tail protein | 99 | YP_009608672.1 |
| ORF49 | 38975 | 39394 | 419 | tail protein | 100 | YP_009322865.1 |
| ORF50 | 39394 | 39789 | 395 | tail protein | 100 | YP_009322866.1 |
| ORF51 | 39786 | 40145 | 359 | tail protein | 100 | YP_009322867.1 |
| ORF52 | 40145 | 40750 | 605 | neck protein | 96 | YP_009322868.1 |
| ORF53 | 40753 | 41259 | 506 | hypothetical protein | 97.6 | YP_009191581.1 |
| ORF54 | 41402 | 41596 | 194 | hypothetical protein | 95.3 | YP_009322870.1 |
| ORF55 | 41633 | 41983 | 350 | immunoglobulin domain-containing protein | 98.3 | YP_224027.1 |
| ORF56 | 41995 | 42279 | 284 | hypothetical protein | 100 | YP_224029.1 |
